# Editorial: Modeling and simulation of cerebrospinal fluid disorders

**DOI:** 10.3389/fbioe.2023.1331170

**Published:** 2023-12-13

**Authors:** Seifollah Gholampour

**Affiliations:** Department of Neurological Surgery, University of Chicago, Chicago, IL, United States

**Keywords:** cerebrospinal fluid (CSF), intracranial compliance (ICC), lumbar interbody fusion (LIF), TRPV4 mRNA, intrathecal drug delivery (ITDD), intracranial pressure (ICP), computational fluid dynamics (CFD), fluid-structure interaction (FSI)

## Introduction

Cerebrospinal fluid (CSF) disorders are known for their considerable complexities in pathophysiology, diagnosis, and treatment. These disorders also pose substantial challenges for physicians, as their common neuroimages and clinical overlaps may be confusing and difficult to diagnose and treat. Therefore, the reliance on precise quantitative indicators becomes paramount, in addition to neurological and physical examinations, and patient clinical histories, to provide a nuanced understanding of these disorders and evaluate treatment outcomes. The emergence of bioengineering computer simulation and modeling stands out as a potent and non-invasive tool, providing a quantitative framework for evaluating CSF disorders (Gholampour, 2018). Recent advances in computer simulation have facilitated the development and application of intricate models, providing valuable insights for the diagnosis, prognosis, and management of CSF disorders. However, despite the progress made, many challenging and controversial points persist in these modeling and simulations. These include determining optimal simulation and modeling parameters (Makin, 2019; Datteri, 2020), ensuring correct model simplifications (Makin, 2019; Datteri, 2020), accurately defining boundary conditions (Gholampour and Fatouraee, 2021), and addressing the time-consuming nature of simulation methods (Gholampour, 2021) that struggle to resemble real physiological conditions accurately. This Research Topic, consisting of seven papers, seeks to provide more in-depth studies addressing these concerns, elucidating the complexities of CSF disorders, and offering insights into the mechanisms underlying these disorders. The goal of this Research Topic is to advance our understanding and improve diagnostic, prognostic, and management tools for CSF disorders.

## Computational model for CSF dynamic analysis and neural simulations

Chlasta et al. and Vandenbulcke et al. concentrated on identifying optimal, facilitated, and cost-effective parameters for modeling and simulating CSF disorders and neural simulations.

Previous studies demonstrated that evaluating CSF dynamics is critical for understanding the complexities in the mechanisms underlying CSF disorders, and it is effective in the diagnosis and treatment of these disorders (Gholampour et al., 2017a; Gholampour et al., 2017b). However, the translation of results from *in vitro* and animal studies to humans is problematic (Hackam and Redelmeier, 2006). *In vivo* studies also face ethical barriers in replicating specific clinical scenarios. Consequently, computational modeling and simulations stand out as precise methods for investigating CSF dynamics, offering a way to bypass the restrictions linked with other approaches. Vandenbulcke et al. in a study titled “*Computational fluid dynamics model to predict the dynamical behavior of the cerebrospinal fluid through implementation of physiological boundary conditions*,” developed a computational framework capable of predicting CSF pressures and velocities across a range of physiological states. This was achieved via a three-dimensional computational fluid dynamics (CFD) model grounded in medical imaging and augmented by physiological conditions as boundary constraints. The model’s predictions were validated against *in vivo* measurements of CSF flow within the subarachnoid space and the cerebral aqueduct. The model was refined to include respiratory influences, resulting in an increase in compliance from 0.17 to 0.51 mL/mmHg. The analysis revealed a significant relationship between outlet-specific compliance and the corresponding CSF outflow. This research extended beyond certain boundaries identified in prior investigations by incorporating elements such as CSF secretion into the lymphatic and venous systems, fluctuations in blood vessel volumes, and compliance within the intracranial and spinal regions. The findings highlighted the influential role of compliance distribution on the orientation of CSF flow and clarified how overall compliance affects the magnitude of pressure oscillations.

Previous studies have shown that one of the primary challenges in conducting computer simulations of CSF disorders, as well as more broadly central nervous system (CNS) disorders, lies in the necessity for large-scale simulations, which face difficulties from numerical, time-consuming, and budgetary perspectives (Datteri, 2020). Chlasta et al. in a study titled “*Neural simulation pipeline: Enabling container-based simulations on-premise and in public clouds*,” introduced a neural simulation pipeline (NSP) with the aim of reducing entry barriers for neural simulations, applicable to both brain networks and biomechanical simulations of the brain. This enhances their feasibility and cost-effectiveness for large-scale computer simulations across diverse computing infrastructures, utilizing the infrastructure-as-code containerization approach. The pipeline was evaluated through the execution of 54 simulations. The results of all simulations were centrally stored and made accessible via a single online storage system, and the experimental data were also processed and stored. They evaluated the NSP using RetNet models of the liquid state machine, which included up to 12,040 neurons and were executed through the general neural simulation system (GENESIS). The authors demonstrated that their Docker-based pipeline enabled the simulations to be developed, tested, and run in either an on-premise or public cloud environment. This study introduced a new simulation management method that simplifies model development and simulation across various execution environments. The findings indicated that this NSP has the potential to lower obstacles for conducting neural simulations, making them more feasible and cost-efficient.

## Application of modeling and simulations in diagnosis and treatment of CSF disorders

Seiner et al., Gholampour et al., White et al. and Kim et al. have utilized non-invasive simulation and modeling in the evaluation of communicating hydrocephalus, normal pressure hydrocephalus (NPH), and disorders related to intrathecal drug delivery (ITDD) injection in CSF flow, as well as in the context of lumbar interbody fusion (LIF) (Gholampour et al., 2022a).

Previous studies highlighted how the dynamics of CSF interact with the variables of ITDD to enhance therapeutic approaches for CNS disorders (Manuel et al., 2023). Seiner et al. in a study titled “*Investigation of human intrathecal solute transport dynamics using a novel in vitro cerebrospinal fluid system analog*,” examined a range of injection parameters used during lumbar puncture, such as the volume and speed of injections, the specific location of injections, and the type of device utilized, assessing their effects on the transport of intrathecal drug to the brain. In the subject-specific *in vitro* modeling constructed, CSF production was initiated at the lateral ventricles of the model, while absorption occurred at the location of the superior sagittal sinus. Using fluorescein as a tracer, the model’s small molecule simulated the drug, tracking its movement throughout the system for 3 hours following lumbar ITDD injections. It was observed that the volume of the flush had a significant influence on the enhancement of solute transport to the brain within 3 hours, with a marked increase of +3.9% ID (*p* = 0.009). Their findings suggested that by adjusting injection parameters and devices, even slightly, lumbar spine ITDD injection techniques can be refined to enhance the efficacy of drug delivery to the brain. Their study offered a foundation for refining guidelines for intrathecal drug administration and further enhancement of ITDD injection methodologies to bolster solute distribution to the brain, which is crucial for treating CNS and/or CFS disorders.

Previous studies showed that intracranial compliance (ICC), represented by the ratio ∆V/∆P, was established as a key metric to evaluate CSF disorders and the recovery behavior of the brain in these disorders (Gholampour, 2023). Gholampour et al. in a study titled “*A new definition for ICC to evaluate adult hydrocephalus after shunting*” proposed a revised definition ICC, termed long-term ICC, using image-based fluid–structure interaction (FSI) simulations (S Gholampour et al., 2022a). They provided brain images from 15 adults with communicating hydrocephalus. Their analysis was conducted before shunt surgery and at eight different phases, continuing until 18 months after shunting. Each phase involved calculating CSF volume (CSFV) and intracranial pressure (ICP), enabling noninvasive ICC calculations. They validated the correctness of the calculated ICP by comparing it with experimental ICP monitoring results. Stability in the values of CSFV, brain volume, and ICP was attained at 12, 15, and 6 months post-shunting, respectively. Findings indicated that the brain required roughly 2 months to adjust to the fast drop in ICP following shunting, potentially due to the brain’s viscous time-dependent component. These noninvasive, long-term ICC measurements revealed a non-uniform CSFV–ICP relationship, with ICC values showing oscillatory increases throughout the shunt treatment. These oscillatory patterns in long-term ICC might mirror clinical fluctuations observed in hydrocephalus patients undergoing shunt procedures. Their new definition of ICC is also valuable for the practical application of ICC as an indicator in the diagnosis and treatment of CSF disorders.

Previous studies showed that the clinical manifestations of NPH and Alzheimer’s disease (AD) can often be indistinguishable, leading to frequent misdiagnosis between these two disorders (Reeves et al., 2020). White et al. in a study titled “*TRPV4 mRNA is elevated in the caudate nucleus with NPH but not in Alzheimer’s disease*,” utilized a comparative genomics modeling and suggested the possibility of distinguishing NPH from AD by identifying mRNA biomarkers closely associated with telomeres. In their study, *postmortem* tissue samples were collected from elderly subjects, providing frozen samples of the caudate nucleus from various groups: seven control individuals, seven subjects diagnosed with NPH, and five patients with AD. The mutation propensity of transient receptor potential cation channel subfamily V member 4 (TRPV4) and other genes in mice, rats, and humans was analyzed by comparing nucleotides of six selected genes, along with one reference housekeeping gene. Their study revealed an increase in the levels of TRPV4 and the mRNA of microtubule-associated tau protein in cases of NPH. While the mRNA expression levels of TRPV4 remained consistent in AD, there was a variance in the expression of amyloid precursor protein and other genes. It was also observed that the TRPV4 gene was located less than 50 megabases from the telomeres in these species, which include mice, rats, and humans. The findings suggested that TRPV4 may serve as a potential connection in the underlying mechanisms of chronic hydrocephalus in older humans (aged 65 years and over) and laboratory rodents of similar age brackets.

Previous studies underscored that finite element simulation has been established as a valuable tool for evaluating lumbar flexibility, movement, and stability, which are important parameters for enhancing the effectiveness of certain surgical procedures designed to treat spinal disorders (Gholampour et al., 2016). Kim et al. in a study titled “*Spinal stability analysis of lumbar interbody fusion according to pelvic type and cage angle based on simplified spinal model with various pelvic indices*” evaluated lumbar flexibility and the stability of spinal fixation in relation to different pelvic anatomies. This assessment involved analyzing the range of motion (ROM) and stress distribution post-LIF using finite element simulations derived from 2D X-ray imagery. Models representative of three pelvic configurations—neutral pelvis (NP), anterior pelvis (AP), and retroverted pelvis (RP)—were chosen based on the pelvic tilt and subjected to LIF. Variations in cage design were introduced by employing different cage angles: 0°, 4°, and 8°. The NP model demonstrated the greatest spinal ROM, whereas the AP model showed increased lumbar lordosis. The NP model also presented greater lumbar flexibility when subjected to complex loading, a reduction of 2.46° on average compared to pure moment loading scenarios. Their findings showed that a 0° cage angle resulted in a limited ROM across all pelvic types. Stress exerted on the vertebrae was minimal using a 0° cage in both NP and AP types. Conversely, the RP type experienced the least stress when a 4° angled cage was implemented. The pelvic anatomy emerged as a more influential factor on ROM than the cage angle. These findings can be of great importance for a neurosurgeon to improve the efficacy of LIF.

## Computational modeling of pulse wave dynamics in cerebral blood vessels

Previous studies have demonstrated the complex interconnection between cerebral blood flow and CSF flow, highlighting their significant impact on CSF disorders (Bothwell et al., 2019; Gholampour et al., 2023). Therefore, studying cerebral blood vessels has an indirect impact on the underlying mechanisms of CSF disorders. Koch et al. in a study titled “*Angiographic pulse wave coherence in the human brain*” developed an innovative computational approach to examine pulse waves in blood vessels using cerebral angiograms that were minimally invasive. They provided the first definitive proof of arteriovenous coupling in the human brain by employing wavelet computational techniques on cerebral angiograms from four patients diagnosed with cerebral aneurysms. They investigated whether employing cross-correlated wavelet reconstruction on human X-ray angiograms could aid in distinguishing distinct vascular pulse waves within the brain, differentiating them into arterial and venous types. Their findings indicated that the reciprocal phase of cardiac frequency within a specific small region of interest in a certain artery could predict the venous structure on a pixel-by-pixel basis, and that these predictions could reconstruct the timing of venous bolus passage. Both predicted pixel clusters from the arteries and veins preserved a complementary phase throughout the bolus travel. Additionally, this research could pave the way for exploring disrupted arteriovenous coupling as an overlooked pathological disturbance in various neurological and CSF disorders associated with mechanical imbalances. The findings also set the stage for future research aimed at delineating cerebrovascular physiology in a living intact human brain and creating an elaborate spatiotemporal “atlas” of arteriovenous coupling during both normal and abnormal conditions.

## Perspectives and conclusion

The collection of seven papers in the Research Topic represents significant progress in leveraging computational models to understand and manage CSF disorders. The focus on refining simulation parameters and enhancing non-invasive diagnostic practices represents significant progress for the practical application of these findings in clinical settings. Studies investigating drug delivery through ITDD and proposing a new definition for ICC offer practical insights for treatment advancements. Genomic modeling shows promise in reducing misdiagnosis in certain CSF disorders. The adoption of finite element simulations underscores the importance of individualized surgical planning, complementing efforts to personalize treatment in spinal disorders. The novel exploration of cerebral pulse wave dynamics highlights an intricate connection between blood flow and CSF, and consequently, CSF disorders, presenting potential avenues for future research.

In conclusion, the seven papers in this Research Topic illuminate the evolving landscape of CSF disorders, emphasizing the pivotal role and transformative potential of computational modeling in elucidating these conditions. The studies underscore the progress made in optimizing parameters, lowering barriers, and enhancing feasibility in simulations of CSF disorders, highlighting the critical role of computational methodologies in modern medicine. These computational approaches not only enhance our understanding of underlying mechanisms but also contribute to refining diagnostic and treatment strategies for individuals affected by these complex disorders, providing valuable insights into the pathophysiology of various CSF disorders. The interdisciplinary synergy showcased here, amalgamating bioengineering, fluid dynamics, neurology, and computational neuroscience, lays the groundwork for future studies that may 1 day unravel the full potential of simulation-based medical introspection and treatment, ultimately leading to more effective treatment strategies.

## Abbreviations

AD, Alzheimer’s disease; AP, Anterior pelvis; CFD, Computational fluid dynamics; CSF, Cerebrospinal fluid; CSFV, Cerebrospinal fluid volume; CNS, Central nervous system; FSI: Fluid–structure interaction; ICC, Intracranial compliance; ICP, Intracranial pressure; ITDD, Intrathecal drug delivery; LIF, Lumbar interbody fusion; NSP, Neural simulation pipeline; GENESIS, General neural simulation system; NPH, Normal pressure hydrocephalus; NP, Neutral pelvis; ROM, Range of motion; RP, Retroverted pelvis; TRPV4, Transient receptor potential cation channel subfamily V member 4.
